# Transgenerational effects of pyriproxyfen in a field strain of *Musca domestica* L. (Diptera: Muscidae)

**DOI:** 10.1371/journal.pone.0300922

**Published:** 2024-03-22

**Authors:** Naeem Iqbal, Nauman Sadiq, Muhammad Nadir Naqqash, Muhammad Usman, Hafiz Azhar Ali Khan, Allah Ditta Abid, Muhammad Sohail Shahzad

**Affiliations:** 1 Institute of Plant Protection, MNS University of Agriculture, Multan, Pakistan; 2 Department of Plant Protection, Ministry of National Food Security & Research, Pakistan; 3 Department of Agronomy, MNS University of Agriculture, Multan, Pakistan; 4 Institute of Zoology, University of the Punjab, Lahore, Pakistan; Beni Suef University Faculty of Veterinary Medicine, EGYPT

## Abstract

*Musca domestica* L. (Muscidae: Diptera) is a human and livestock pest especially in tropical and sub-tropical areas. Different insecticides have been used to control this pest that pose serious harmful effects on humans and the environment. The current study was planned to investigate the effects of two concentrations (LC_25_ and LC_50_) of pyriproxyfen on biological and population parameters of a field strain of *M*. *domestica*. The exposed parents (F_0_) and their progeny (F_1_) were studied to examine the transgenerational effects. The results indicated that preadult duration was higher in control (13.68 days) compared to LC_50_ treated individuals (12.44 days). The male and female longevity was relatively lower in the LC_25_ treated population i.e. 24.62 and 26.62 days, respectively. The adult pre-oviposition period (APOP) and total pre-oviposition period (TPOP) values were higher in the LC_25_ treated individuals than those of control. Moreover, oviposition days and fecundity were reduced in the treated individuals as compared to the control treatment. A gradual decrease in the net reproductive rate (*R*_*0*_) was observed (8.46–14.07 per day) while the value of *R*_*0*_ was significantly higher in control. The results suggested that pyriproxyfen can be effectively utilized and incorporated in the management programs of *M*. *domestica*.

## Introduction

The common housefly, *Musca domestica* L. (Muscidae: Diptera) is a major pest of humans and animals in the tropical and sub-tropical areas of the world. It serves as a mechanical vector for numerous pathogenic bacteria and other microorganisms [1, 2]. Some of the important diseases spread by house fly include salmonellosis, shigellosis, and cholera [3]. They are also involved in rickettsial and viral syndromes including helminthic (e.g. roundworms, hookworms, pinworms, and tapeworms) and protozoan pathogens (e.g. amoebic dysentery) [4]. All these pathogenic microorganisms are spread when house flies defecate and/or regurgitate on the food items [5].

House flies are mainly controlled by the use of highly toxic and residual insecticides. However, the injudicious use of insecticides to control house fly populations has led to the development of resistance to most of the insecticides. *M*. *domestica* was the first insect in which resistance was reported against organochlorine (DDT) [6]. Later, it developed resistance to organophosphates (45.4–62.5 folds to dichlorvos), carbamates (>290-fold to propoxur) and many other insecticides from different groups [7–9]. In addition, the unwise use of pesticides caused serious harmful effects on the environment, humans and other non-target organisms. Consequently, the use of non-conventional insecticides like insect growth regulators (IGRs) has received considerable attention in recent years, especially in the indoor environment [10, 11]. It has been well documented that IGRs are comparatively safer to the environment and mammals and break down easily in nature. These features make them viable alternatives to traditional insecticides for house fly control [12].

Pyriproxyfen is an IGR which acts on the endocrine system of insects by mimicking the naturally occurring juvenile hormone. It hinders molting and adult formation in insects, and can be an important component of Integrated Pest Management program [13]. It has been found very effective against a wide variety of insects belonging to Lepidoptera, Hemiptera and Diptera orders [14–16]. Pyriproxyfen lowers female mosquito fecundity by interfering with egg maturation or transferring eggs to the larval environment [17]. A new method, enhanced sterile insect technique (SIT), involves dusting radiation-sterilized males with pyriproxyfen before release. In semi-field conditions, the deployment of pyriproxyfen-dusted cloths within test huts led to a 96% decrease in indoor pests (mosquitoes) compared to control [18]. Though, the effects of pyriproxyfen are studied on larval stages of *M*. *domestica* [19, 20], but little is known about the sublethal effect of pyriproxyfen on adults. The studies involving lethal and sublethal effects of pyriproxyfen on the demographics and biological parameters of *M*. *domestica* after exposure to adults are lacking. The sublethal exposure induces variation in the P450s [21, 22] which in turn increases pesticide metabolism and may result in pest outbreak [23]. Generally speaking, insects receiving lethal and sublethal exposure to insecticides usually result in their mortality, altered physiology and biological traits or change in their feeding behavior [20]. Lethal and sublethal exposures may also result in pest’s population suppression by lengthening developmental time, reducing survival and fecundity. Therefore, the changes induced by lethal and sublethal doses should be taken into account for pest management [24]. The current study was aimed to evaluate transgenerational effects of pyriproxyfen in a field strain of *M*. *domestica*. The transgenerational analysis will be helpful to explore hormetic effect (low dose stimulation) of pyriproxyfen which may lead to increase biotic potential in exposed insect pest populations. Age-stage, two sex life table was utilized to compare the exposed and unexposed progeny of *M*. *domestica*.

## Materials and methods

### Insecticides and *Musca domestica* strain

A commercial formulation of pyriproxyfen 10.8 EC (Syngenta Pakistan) was purchased from a local pesticide market (Multan, Pakistan) to use in bioassays.

Adults of *M*. *domestica* were collected from an agricultural field (30.163458°N, 71.441871°E) in Multan, Punjab, Pakistan. These adults were shifted to mesh cages (30 × 30 × 60 cm) and placed in the laboratory at 25 ± 2°C temperature, 60±5% relative humidity and 16:8 light-to-dark ratio. They were fed on 20% sugar solution on a cotton plug. Plastic cups (5 cm high, top Ø 8.3 cm, bottom Ø 7.2 cm) containing larval diet were placed in the cages for egg laying. The larval diet consisted of rice husk, wheat bran, sucrose, milk powder and yeast in ratio of 40: 10: 3: 1: 1 along with small quantity of water [20].

### Lethal concentration estimation

The acute toxicity of pyriproxyfen to *M*. *domestica* was tested using a food-incorporated bioassay method developed by Subaharan et al. [25] with little modification. Serial dilutions (ranging from 1 μg/ml to 10 μg/ml) of pyriproxyfen were prepared in 20% sugar solution [20]. Each concentration was replicated thrice and 10 adult *M*. *domestica* were released in each replication. The bioassay was conducted in mesh cages at 25 ± 2°C temperature, 60±5% relative humidity and 16:8 light-to-dark ratio. A cotton plug was dipped in a specific concentration and placed inside the mesh cage as adult food. The adult mortality was recorded after 72 h of the treatment.

### Transgenerational studies

The transgenerational studies were conducted using the methodology of Ghramh et al. [26]. Briefly, LC_25_ and LC_50_ concentrations of pyriproxyfen were prepared in 20% sugar solution and 50 adults (4–5 days old) were exposed to each concentration. The similar numbers of adults were also kept as control. After 72 hours of exposure, a larval medium or egg laying material (as described above) was provided to the treated adults (F0) or the control group in plastic cups (5 cm high, top Ø 8.3 cm, bottom Ø 7.2 cm). The numbers of eggs laid in each cup were counted on daily basis. For transgenerational studies, 50 eggs from each group were separated very carefully and placed in new cups containing larval diet. The egg hatching in each group was observed on daily basis. After hatching, the larvae were reared in the same groups in small plastic boxes (4×15×10 cm) until adult eclosion at 25± 2°C under a 16:8 h light–dark photoperiod and 60±5% relative humidity. After adult emergence (F1), pre-oviposition period, oviposition period and fecundity were determined following the methodology of Ghramh et al. [26].

### Statistical analyses

The probit analysis was used to estimate lethal concentrations (LC_25,_ LC_50_), their fiducial limits (FL), the slope and other related parameters using POLO-PLUS^®^ software program (LeOra Software 2003, Inc., Berkeley, CA). To develop a life table, the data were evaluated by using the theory of age-stage, two sex life table. The population parameters, included in this study were the age-stage specific survival rates (*s*_*xj*_, where *x* is age and *j* is stage), net reproductive rate (*R*_0_), the mean generation time (*T*), finite rate of increase (λ。) age-specific survival rate (*l*_*x*_), and intrinsic rate of increase (*r*). Additionally, based on the population parameters, other parameters viz. age-specific fecundity (*m*_*x*_), age-stage specific life expectancy (*e*_*xj*_), and age-specific maternity (*l*_*x*_*m*_*x*_) were also calculated [27–31].

For assessment of reproductive/biotic potential in a better way, total pre-oviposition period (TPOP), adult pre-oviposition period (APOP), reproductive days (*R*_*d*_) (i.e., the number of days that adult produced offspring), reproductive value (*v*_*xj*_), were computed using the computer program TWOSEX-MSChart [29]. The variances and standard errors of the population parameters were estimated using the bootstrap procedure with 100,000 random resampling and difference of population parameters between control and insecticide treatment groups was compared by using the paired bootstrap test based on the confidence intervals of differences implemented in TWOSEX-MSChart [29]. All graphics were created using SigmaPlot 12.0 (Systat Software Inc., San Jose, CA, USA).

The population parameters were estimated by following equations [27–31]:

The value of *l*_*x*_*m*_*x*_ was calculated by using the equation [29]:

$$\overset{\infty }{\underset{x=0}{\sum }}e^{-r(x+1)}l_{x}m_{x}=1$$

Age-stage specific survival can be calculated using this statistical formula [27]:

$$lx=\overset{k}{\underset{j=1}{\sum }}S_{xj}$$

*R*_*o*_ was calculated by using the equation [29, 30]:

$$R_{0}=\overset{\infty }{\underset{x=0}{\sum }}l_{x}m_{x}$$

The equation to determine the *T* value was [29]:

$$T=(In\;R_{o})/r$$

The value of *r* was estimated by:

$$\sum_{x=0}^{\infty }e^{-r(x+1)}l_{x}m_{x}=1$$

Finite rate of increase was calculated by:

$$\lambda_{o}=e^{r}$$

The value *e*_*xj*_ was determined by [30]:

$$e_{xj}=\overset{\infty }{\underset{i=x}{\sum }}\;\overset{k}{\underset{y=j}{\sum }}s_{iy}^{\prime }$$

*V*_*xj*_ value was determined with equation [31]:

$$v_{xj}=\frac{e^{-r(x+1)}}{S_{xj}}\overset{\infty }{\underset{i=x}{\sum }}e^{-r(x+1)}\overset{k}{\underset{y=j}{\sum }}S_{iy}^{\prime }f_{iy}$$

## Results

### Lethal concentration estimation

The field strain of *M*. *domestica* was exposed to concentrations of pyriproxyfen, and LC_50_ and LC_25_ values were calculated based on the data collected after 72 h of exposure to various concentrations. LC_10_ and LC_25_ values for the pyriproxyfen were 0.83 and 1.94 μg/ml, respectively. Whereas, the LC_50_ value of pyriproxyfen against *M*. *domestica* was 4.99 μg/ml (Table 1).

**Table 1:** Toxicity analysis of pyriproxyfen against *Musca domestica* after 72 h of exposure.

Sr. No.	Lethal Concentration	Doses (μg/ml)	Fiducial limit	χ^2^	DF	Slope
**1**	LC_10_	0.83	0.27–1.55	2.15	4	1.72±0.28
**2**	LC _25_	1.94	0.90–3.06	2.15	4	1.72±0.28
**3**	LC _50_	4.99	3.21–6.85	2.15	4	1.72±0.28

**χ**^**2**^ = Chi Square, DF = Degree of Freedom

### Basic parameters

The effects of concentrations of pyriproxyfen on the field strain of *M*. *domestica* are presented in Table 2. The pre-adult duration was much higher in the control treatment (13.68) while it was lower in the LC_25_ (12.50) and LC_50_ treated (12.44) individuals. The female longevity in the control treatment was higher those of LC_25_ and LC_50_ treated individuals. Similarly, male longevity was significantly higher (27.89) in the control group and significantly lower (24.62) in both the LC_25_ and LC_50_ groups.

**Table 2:** Transgenerational effect of pyriproxyfen on different life table parameters of *Musca domestica* (F1).

Parameters	Control	LC_25_	LC_50_
Pre-adult duration	13.68± 0.07a	12.50± 0.07b	12.44± 0.07b
Female longevity	28.80± 0.78a	26.41±0.75a	27.56± 0.91a
Male longevity	27.89±0.54a	24.62± 0.53b	24.62± 0.53b
APOP	1.87±0.09b	5.50±0.22a	2.56±0.29b
TPOP	18.26± 0.26b	19.75 ±0.30a	18.56±0.41b
Oviposition days	7.93±0.37a	5.25±0.39b	5.00±0.00b
Fecundity	61.25 ± 1.24a	57.67± 1.79a	47.00±1.36b

TPOP: Total pre-oviposition period of female counted from birth, APOP: Adult pre-oviposition period of female counted from adult eclosion from pupae.

Means in the same row followed by the same letter are not significantly different (P > 0.05) using bootstrap test.

The adult pre-oviposition period (APOP) values were significantly higher (5.50 days) in the population treated with LC_25_, while the value of APOP in the control group was significantly lower (1.87 days). The total pre-oviposition period (TPOP) of the progeny of population treated with LC_25_ was significantly higher (19.75) compared to control (18.26). The oviposition days were significantly higher in the control population (7.93). While, the group that was treated with LC_25_ and LC_50_ had a significantly lower number of oviposition days. The fecundity was significantly higher in the control group, whereas the fecundity was significantly lower in individuals that had been treated with LC_50_ (Table 2).

### Population parameters

Transgenerational effect of pyriproxyfen on the population parameters is shown in Table 3. The intrinsic rate of increase (*r*) in the control was significantly higher (0.12/day) while it was lower in LC_50_ treated individuals (0.09/day). The net reproductive rate (*R*_*0*_) was significantly higher in the control group (17.3 offspring/day) and significantly lower in the LC_50_ treated individuals (8.46 offspring/day). The values of mean generation time in control and treated individuals were statistically at par. The LC_25_ treated group had comparatively higher mean generation time (*T* = 22.84 days) than the control (*T* = 21.23 days) and LC_50_ treated groups (*T* = 21.45 days). Lambda (*ʎ*) values indicated that finite rate of increase (per day) was also not significantly affected (Table 3).

**Table 3:** Transgenerational effect of pyriproxyfen on population parameters of *Musca domestica*.

Parameters	Control	LC_25_	LC_50_
*r* (per day)	0.12±0.01a	0.11±0.01a	0.09±0.01b
*R*_*o*_ (per day)	17.30± 3.83a	14.07± 3.84a	8.46±2.54b
*T* (days)	21.23±0.43a	22.84±0.38a	21.45± 0.44a
λ。(per day)	1.13±0.01a	1.12±0.01a	1.10±0.01a

*r =* The intrinsic rate of increase (per day); *ʎ* = Limiting rate of growth (per day)

*R*_*0*_ = The net reproductive rate (offspring/individual); *T* = The mean generation time (days).

Means in the same row followed by the same letter are not significantly different (P > 0.05) using bootstrap test.

### Age-stage specific maternity (*l*_*x*_*m*_*x*_)

**Fig 1:**
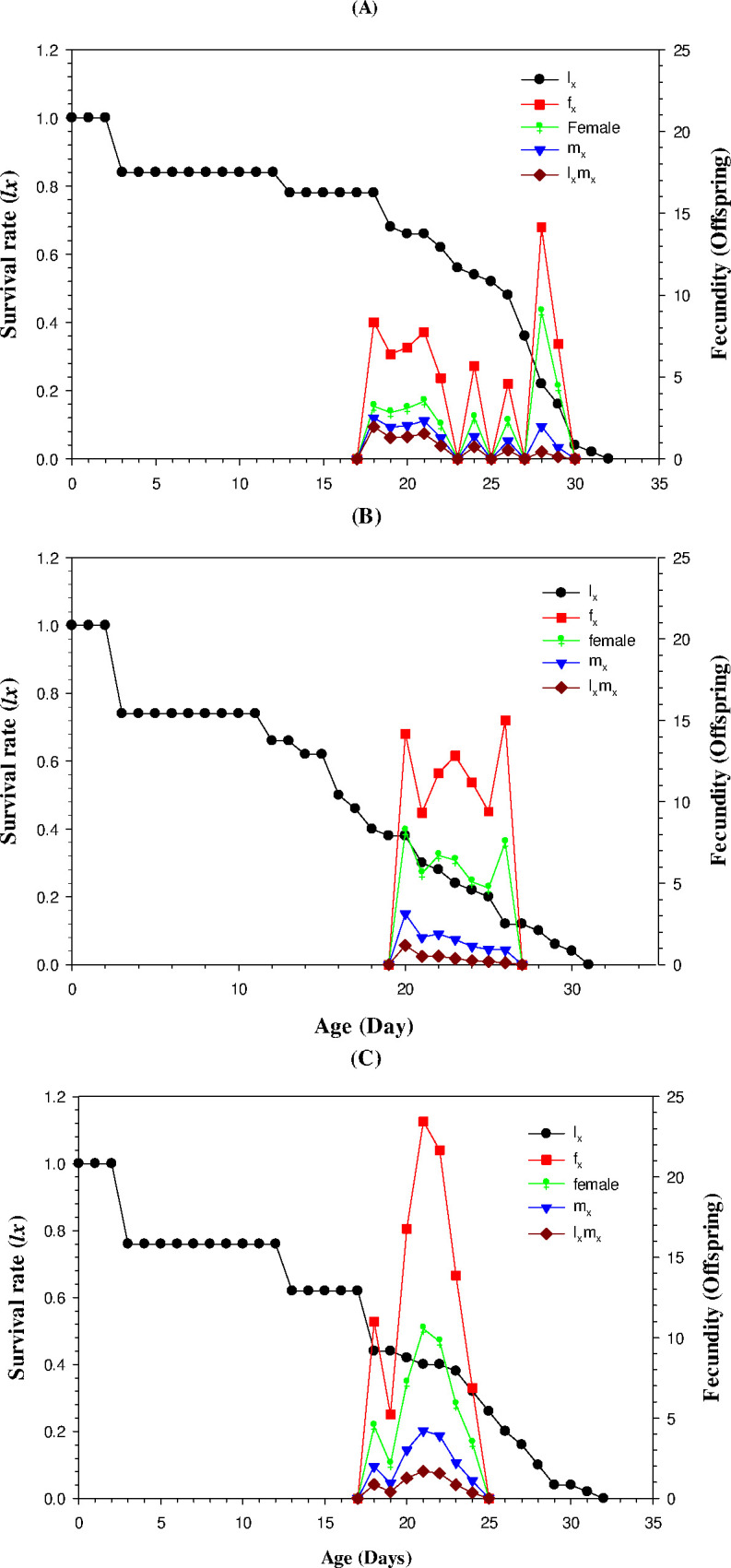
Survival rate (*l*_*x*_), maternity rate (*m*_*x*_) and their product (*l*_*x*_*m*_*x*_) in the progeny of pyriproxyfen treated adults; A) Control B) LC_25_ C) LC_50_.

The results of age-stage specific maternity (*l*_*x*_*m*_*x*_) indicated that the fecundity (*f*_*x*_) was at its highest point (23/day) in 20 days old female and then decreasing trend was observed from day 20 to day 24. The age-stage specific survival rate (*l*_*x*_) also decreased over time and it was around 30 days in LC_25_ treated population and 32 days in control. Age-specific fertility (*m*_*x*_) was 4.32 days in the LC_50_-treated individuals and 2.60 days in the control treatment. Age-specific maternity (*l*_*x*_*m*_*x*_) was maximum (2.08 offspring/day) in the control group while significantly less in LC_25_ treated individuals (1.26 offspring/day) (Fig 1).

### Age-stage specific survival rate (*S*_*xj*_)

**Fig 2:**
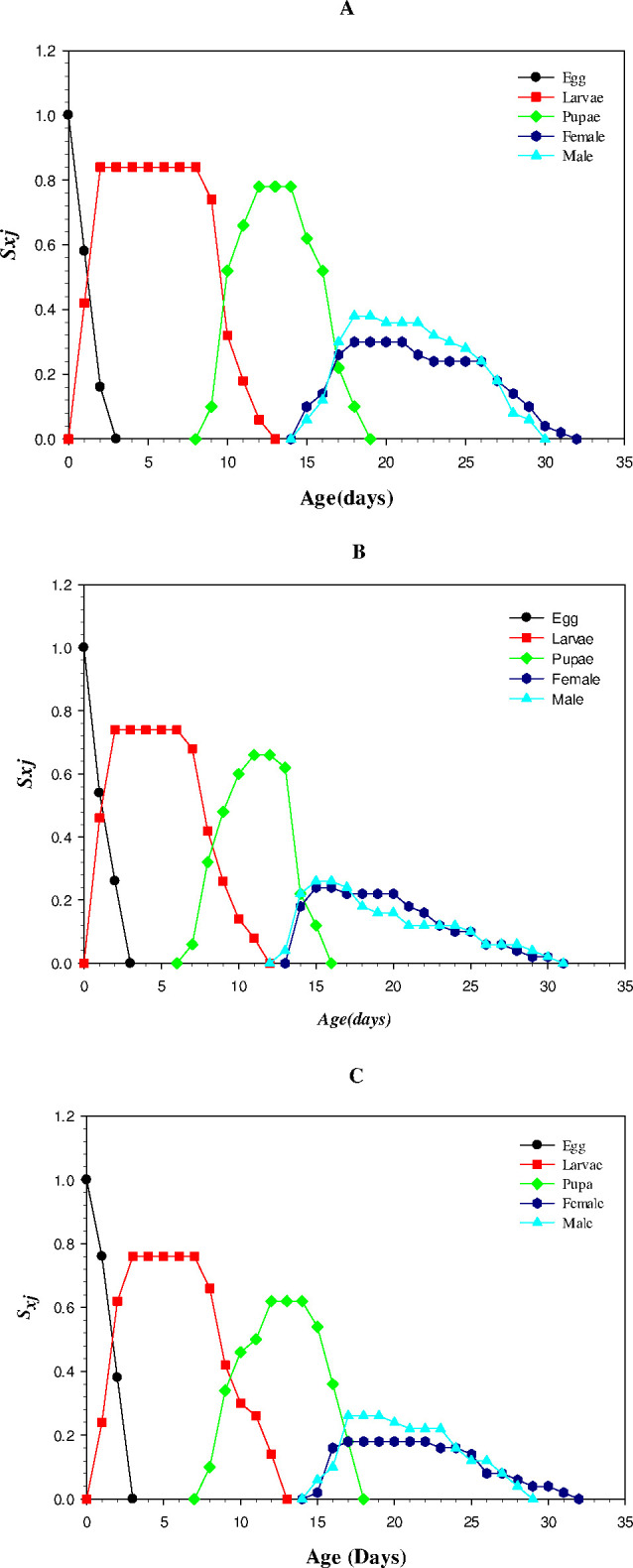
Age stage-specific survival of *Musca domestica* in treated and control groups A) Control B) LC_25_ and C) LC_50_.

Age-stage survival rate (*S*_*xj*_) was higher for larvae in the control group (13.25 days) while it was lower in the LC_25_ treated group (11.99 days). The pupal survival was also higher in control compared to treated individuals. The male age stage-specific survival was higher in the LC_25_ treated individuals than those of LC_50_ treated individuals. For females, *S*_*xj*_ value was higher in LC_50_ treated individuals (32 days) while it was lower in the LC_25_ treated individuals (31 days) (Fig 2).

### Age-stage-specific life expectancy (*e*_*xj*_)

**Fig 3:**
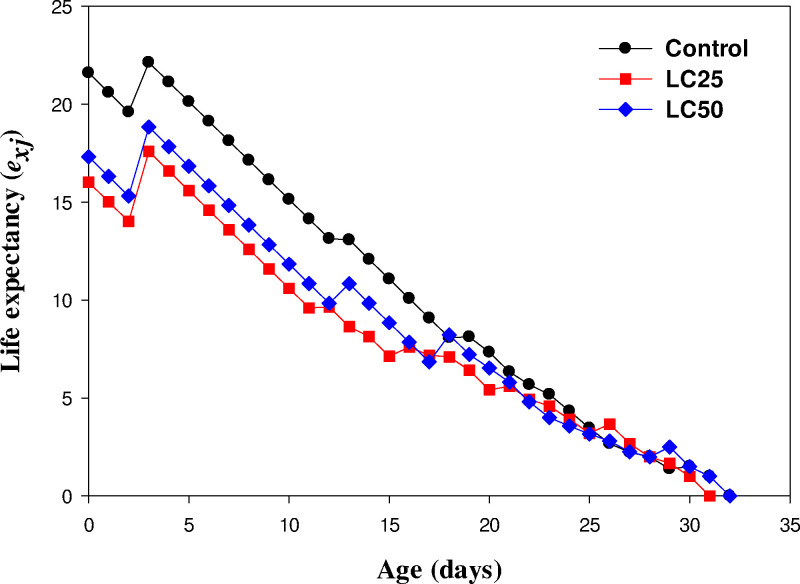
Life expectancy *(e*_*xj*_*)* of *Musca domestica* in control, LC_25,_ and LC_50_.

The age-stage-specific life expectancy (*e*_*xj*_) calculates an individual’s post-age-x life expectancy based on the age-stage, two-sex life table. The life expectancy (*e*_*xj*_) was the highest on day 3 and then gradually declined in all groups. The values of *e*_*xj*_ were higher for control individuals (33.03 days) followed by LC_25_ (31.94 days) and LC_50_ (30.91 days) treated individuals (Fig 3).

### Age-specific maternity (*V*_*xj*_)

**Fig 4:**
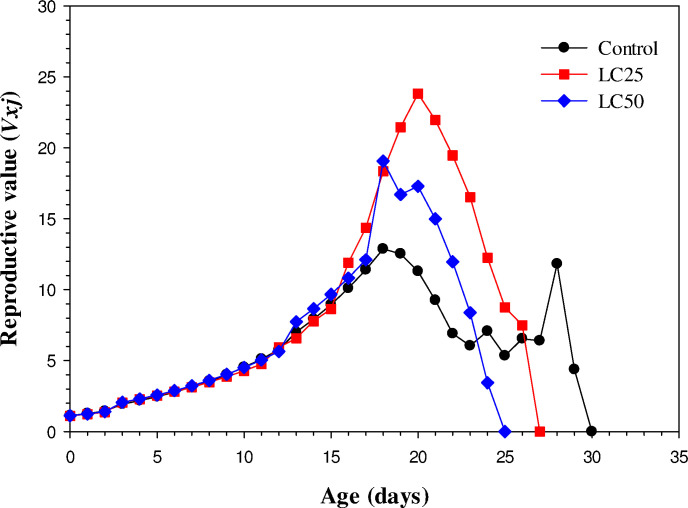
Reproductive value *(V*_*xj*_*)* of *Musca domestica* in control, LC_25,_ and LC_50_.

It is the age-specific maternity or reproductive value (*v*_*xj*_) that contributes to future population growth for individuals of stage j and age x. The control treatment showed the lowest peak of *v*_*xj*_ on day 18 which was decreased to 0 by day 30. The highest peak was observed in the LC_25_ treated population on day 19 compared to other treatments (Fig 4).

## Discussion

Pyriproxyfen is one of the most commonly used Insect Growth Regulator (IGR) against a broad range of insect pests. The current study aimed to explore the sub-lethal effects of pyriproxyfen on the biological and population parameters of *M*. *domestica*. The results indicated that pyriproxyfen significantly reduced preadult duration, male and female survival rate, fecundity and net reproductive rate in treated populations. Current study revealed higher preadult duration in the control population compared to LC_25_ and LC_50_ treated populations. Similar results were obtained by Iftikhar et al. [14] and Mahmoudvand et al. [32] who reported decline in preadult duration in *Plutella xylostella* and *Hippodamia convergens*. Richardson & Lagos [33] reported reduced developmental time in 1^st^ and 4^th^ nymphal instar of *Aphis glycines* as a result of pyriproxyfen exposure. The results of the present study such as toxicity values, response of biological traits and life table parameters after exposure to pyriproxyfen varied from the previous study on *M*. *domestica* [20]. The most probable reason for these variations could be due to differences in geographical origins of strains tested, their susceptibility to insecticides, bioassay conditions and chemicals used in the present and previous studies.

Male and female longevity was low in the treated population while it was higher in control population. A similar trend was observed by Steigenga et al. [34] in *Bicycles anynana* and by Rahmani and Bandani [35] in *H*. *variegata* as a result of sublethal concentrations of pyriproxyfen and thiamethoxam, respectively. Khan [20] also reported decreased longevity of females of an insecticide susceptible strain of *M*. *domestica* when larvae were subjected to sublethal doses of pyriproxyfen. Two other studies reported reduction in female longevity in *P*. *xylostella* and *H*. *convergens* [14, 15].

The sublethal doses of insecticide induce changes in APOP and TPOP values [15]. APOP and TPOP values were considerably greater in the treated population while these values were significantly lower in the control. These findings are in accordance with the findings of Lee et al. [36] and Alizadeh et al. [15]. Alizadeh et al. [15] and Huang et al. [37] also reported increased APOP and TPOP in *P*. *xylostella* as a result of sublethal exposure of pyriproxyfen. Oviposition days were reduced in treated population and the same reduction was observed in *Spodoptera litura* [38]. Fecundity was also decreased in treated populations compared to control, and these variations are confirmed by Alizadeh et al. [15]. Mahmoudvand et al. [32] also reported that sublethal doses of pyriproxyfen reduce the fecundity of *P*. *xylostella*. Khan [20] reported that sublethal exposure of pyriproxyfen to the larval stage of *M*. *domestica* significantly reduces fecundity. These studies showed that pyriproxyfen negatively affected the biological parameters. In addition to this, population parameters are also affected by the sublethal exposure of pyriproxyfen [39].

Net reproductive rate is an important parameter that helps in population determination. The net reproductive rate (*R*_*0*_) was reduced in the treated population. These results are comparable to the previous findings of Mahmoudvand et al. [32] and Shah et al. [39] who reported that the net reproductive rate was reduced in *M*. *domestica* due to sublethal exposure to pyriproxyfen. Reduced net reproductive rate was observed as a result of sublethal exposure of pyriproxyfen to *Anopheles gambiae* [40]. The result of the present study reveals that mean generation time (*T*) was increased in treated population and its value was lower in control. The current findings are consistent with the findings of many previous studies conducted to evaluate the sublethal effects of pyriproxyfen on mean generation time in different insects [15, 41]. Khan [20] reported that there was no significant increase in mean generation time when *M*. *domestica* larvae were treated with the sublethal doses although a slight increase in value was observed in the treated individuals in the present study. The finite rate of increase (*ʎ*) was decreased in the treated population and these findings were verified by previous studies [32]. The decrease in value of *ʎ* was also studied in *Oxycarenus hyalinipennis* by Naeem et al. [42]. Iftikhar et al. [14] also reported a similar decline in *ʎ* while studying the effect of sublethal doses of pyriproxyfen on *H*. *convergens*. These sublethal effects of pyriproxyfen on adults of *M*. *domestica* could be due to its negative effects on hormone production which affect growth and reproduction [43]. Results of current experiment suggest that pyriproxyfen can be effectively used to control *M*. *domestica* populations in different habitat due to lack of hormesis effects [20].

## Conclusion

The present studies showed that the basic parameters (TPOP, APOP, oviposition days, fecundity, male and female longevity) and population parameters (*r*, *R*_*0*_, λ。, *T*) were negatively affected when adults of *M*. *domestica* were exposed to concentrations of pyriproxyfen. The mean generation time (*T*) was higher in the treated population compared to the control population. The current results showed that *M*. *domestica* can be controlled by pyriproxyfen. Based on this data, field trials should be conducted to further ensure the potential of pyriproxyfen against *M*. *domestica*.

## Supporting information

S1 Data (ZIP)
